# Improved electrochemical properties of LiNi_0.91_Co_0.06_Mn_0.03_O_2_ cathode material via Li-reactive coating with metal phosphates

**DOI:** 10.1038/s41598-017-07375-6

**Published:** 2017-08-02

**Authors:** Kyoungmin Min, Kwangjin Park, Seong Yong Park, Seung-Woo Seo, Byungjin Choi, Eunseog Cho

**Affiliations:** 1 Platform Technology Lab, Samsung Advanced Institute of Technology, 130 Samsung-ro, Suwon, Gyeonggi-do 16678 Republic of Korea; 2 Energy Lab, Samsung Advanced Institute of Technology, 130 Samsung-ro, Suwon, Gyeonggi-do 16678 Republic of Korea

## Abstract

Ni-rich layered oxides are promising cathode materials due to their high capacities. However, their synthesis process retains a large amount of Li residue on the surface, which is a main source of gas generation during operation of the battery. In this study, combined with simulation and experiment, we propose the optimal metal phosphate coating materials for removing residual Li from the surface of the Ni-rich layered oxide cathode material LiNi_0.91_Co_0.06_Mn_0.03_O_2_. First-principles-based screening process for 16 metal phosphates is performed to identify an ideal coating material that is highly reactive to Li_2_O. By constructing the phase diagram, we obtain the equilibrium phases from the reaction of coating materials and Li_2_O, based on a database using a DFT hybrid functional. Experimental verification for this approach is accomplished with Mn_3_(PO_4_)_2_, Co_3_(PO_4_)_2_, Fe_3_(PO_4_)_2_, and TiPO_4_. The Li-removing capabilities of these materials are comparable to the calculated results. In addition, electrochemical performances up to 50 charge/discharge cycles show that Mn-, Co-, Fe-phosphate materials are superior to an uncoated sample in terms of preventing capacity fading behavior, while TiPO_4_ shows poor initial capacity and rapid reduction of capacity during cycling. Finally, Li-containing equilibrium phases examined from XRD analysis are in agreement with the simulation results.

## Introduction

There has been increasing demand for lithium ion batteries (LIBs) for application in electric devices such as mobile phones and electrical vehicles. To achieve high energy density and long-term cyclability in LIBs, the use of transition metal (TM)-based oxide cathode materials could be an ideal option because their optimal composition can provide large capacity, low manufacturing cost, and great rate capability^1–4^. Nickel-rich nickel-cobalt-manganese oxide (termed Ni-rich NCM) is a class of promising materials that can satisfy those needs, but they suffer from several types of degradation behaviors such as phase transformation and gas generation^5–7^.

To mitigate degradation behaviors in layered oxide cathode materials thus enhancing the electrochemical performance, the surface modification method, *i.e*., surface coating, has been suggested to provide a physical barrier at the surface of the cathode and prevent the direct contact between active materials and electrolytes. For example, many metal phosphate (MP) materials are suggested as effective coating materials such as MPO_4_ (M = Al, Fe, Ce, and Sr)^8, 9^, Ni_3_(PO_4_)_2_^10^, Mn_3_(PO_4_)_2_^11^, M_3_(PO_4_)_2_ (M = Zn and Mg)^12^, and Zr-phosphate ^13^ for cathode materials such as LiCoO_2_ (LCO), LiNi_0.9_Co_0.1_O_2_, LiNi_0.8_Co_0.15_Al_0.05_O_2_ (NCA), LiNi_0.6_Co_0.2_Mn_0.2_O_2_, and LiNi_0.8_Co_0.15_Mn_0.05_O_2_. In spite of their effectiveness in improving the capacity retention rate, this type of coating approach in principle has drawbacks: 1) Li ion diffusion during electrochemical cycling can be impeded, 2) residual Li still needs to be washed, which requires an additional step during synthesis, and 3) this process can degrade battery performance^14–16^. Li impurities, which are residues on the surface of the cathode formed after initial synthesis, are a major source of gas generation, resulting in swelling behavior inside the battery pack^14, 17^. The excessive amount of Li used as a prerequisite for achieving sufficient capacity with Ni-rich cathode materials is a source of these impurities^17, 18^. Previous studies demonstrated that residual Li (such as LiOH and Li_2_CO_3_) can initiate the decomposition reaction with electrolytes at the interface, leading to the evolution of gas species such as CO_2_, O_2_, N_2_, and CO^14–16^.

Application of Li-containing phosphates such as LiNiPO_4_^19^, Li_3_V_2_(PO_4_)_3_^20^, and LiAlTi(PO_4_)_3_^21^ coating materials has been recommended to alleviate the problem of constricted Li ion diffusion, and they have been shown to improve the rate capability and prevent capacity fading during cycling. Another approach has been suggested to eliminate the two concerns simultaneously, *i.e*., finding coating materials that can directly react with residual Li so that they can be transformed to Li-containing phases and in parallel the amount of Li residue is reduced. For example, Kim *et al*. suggested using Co_3_(PO_4_)_2_ as a Li-reacting coating material that can be converted to LiCoPO_4_ during annealing^16^. AlPO_4_ was also demonstrated to be functional from this perspective by forming Li_3_PO_4_ and LiAlO_2_ after its reaction with Li residues^22^. Jo *et al*. proved that phosphoric acid (H_3_PO_4_) can be changed to Li_3_PO_4_ via a Li-reactive mechanism^23^. However, only a few attempts have been made with this concept, which makes it difficult to choose the optimal MP coating materials for this purpose. It is worth noting that a computational approach has been successfully applied by Wolverton’s group to search for optimal coating materials to scavenge hydrofluoric acid (HF)^24, 25^ and interfacial stability between coating and cathode materials^26^. In this regard, the computational aid is promising for examining and screening MP materials that are highly reactive to Li residues and can form subsequent Li-containing equilibrium phases. The amount of Li residue increases for Ni-rich cathode materials; hence, more surface area is covered with residual Li^17^. Therefore, MP materials are likely to first react with residual Li and form Li-containing structures; remaining unreacted materials still can function as coating material by blocking the direct exposure of the cathode material to the electrolyte.

In this study, we implement a computational framework to propose the optimal MP coating material for removing residual Li from the surface of Ni-rich layered oxide cathode material LiNi_0.91_Co_0.6_Mn_0.3_O_2_ (NCM) by employing first-principles calculations on 16 MP materials. The results are validated with experiments measuring the reduction in the amount of Li residue after applying the coating materials. We also perform electrochemical cycling tests to clarify which of the coating materials is more effective in preventing capacity fading. Finally, the equilibrium phases obtained from experiment are compared to those from the phase diagrams obtained by calculation.

## Results and Discussion

### Design chart and analysis

**Figure 1:**
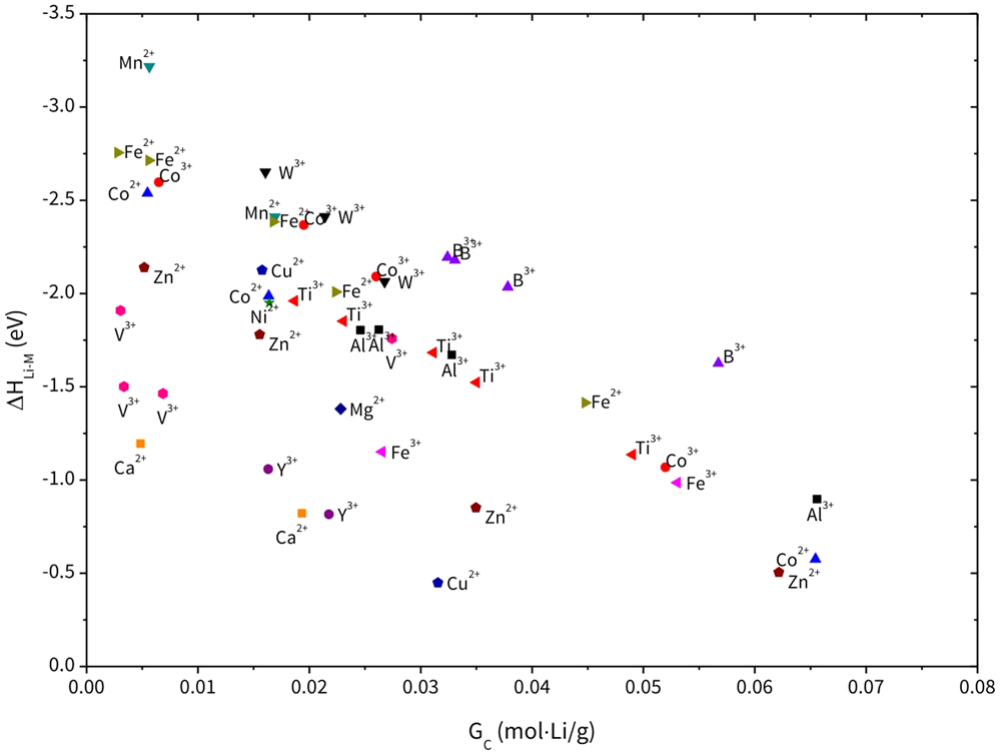
Overall design chart for the reaction between metal phosphate and Li_2_O.

The overall reaction behavior between MP and Li_2_O in terms of reaction enthalpy (ΔH_Li-M_). and gravimetric capacity (G_C_) is shown in Fig. 1 (The complete list of reaction equations is shown in Table S.2 in SI). Each MP material has several possible reactions depending on the amount used. For example, Fe_3_(PO_4_)_2_ can undergo five reactions at various molar ratios from 0.12 to 2 relative to Li_2_O. Among the possible reactions with one material, it is energetically preferable when a larger weight of coating materials is applied. Hence, this relation complicates finding the optimal coating material holding both strengths, *i.e*., the largest reaction energy and the least weight.

To provide comprehensive understanding of the performance of each MP material, we further analyzed the design chart by extracting data and sorting them with respect to their competencies. Two types of data analysis were performed, *i.e*., the descending order of ΔH_Li-M_ and G_C_ values and their corresponding G_C_ and ΔH_Li-M_ values (denoted G_C_′ and ΔH_Li-M_′), respectively. The descending order means that the results are sorted according to the largest value among several possible reaction equations containing each material. Meanwhile, corresponding values (*e.g*., ΔH_Li-M_′) indicate ΔH_Li-M_ of the reaction when sorted in descending order of G_C_, and *vice versa*. The complete list is shown in Table S.3.

**Figure 2:**
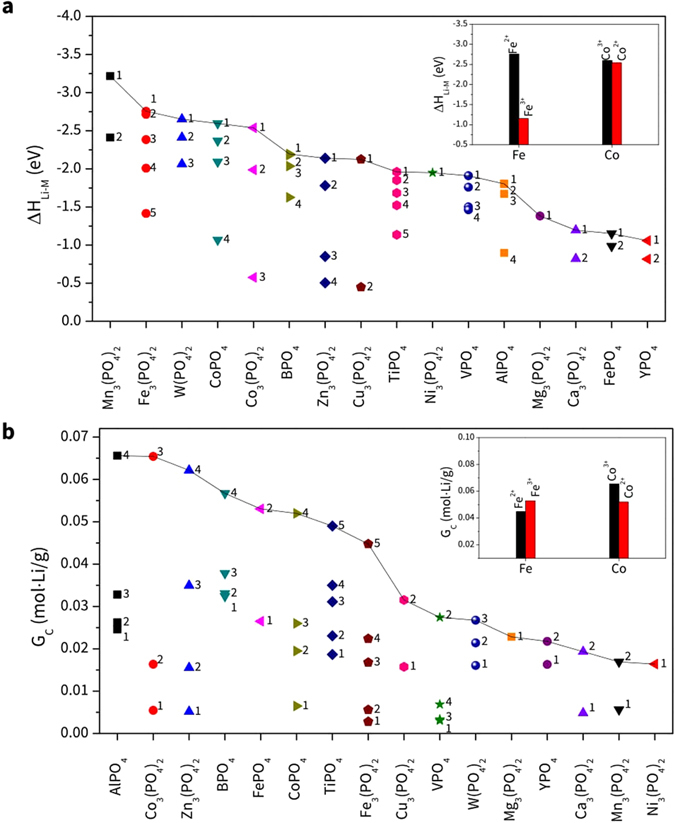
The descending orders of (**a**) ΔH_Li-M_ and (**b**) G_C_, and (inset) comparison between materials having the same metal element for the reaction between metal phosphate and Li_2_O. Each number in (**a**) and (**b**) denotes the reaction equation number.

First, the descending order of ΔH_Li-M_ obtained in Fig. 2a exhibits that Mn_3_(PO_4_)_2_ is the most energetically preferable, followed by Fe_3_(PO_4_)_2_, W(PO_4_)_2_, CoPO_4_, Co_3_(PO_4_)_2_, etc. The most preferable reaction from each material is shown in equation number 1 in Table S.2. In terms of G_C_ (shown in Fig. 2b), AlPO_4_ is calculated to be the most efficient followed by Co_3_(PO_4_)_2_, Zn_3_(PO_4_)_2_, BPO_4_, FePO_4_, etc.

Several significant findings can be addressed based on the above analysis. First, the MP materials positioned at higher ranks in terms of ΔH_Li-M_ generally exhibit poor efficiency, *i.e*., they require a larger weight to achieve that reaction. For example, the most Li-reactive material (Mn_3_(PO_4_)_2_) is placed at 11^th^ in order of G_C_′ and at 15^th^ in the descending order of G_C_. However, it is worthwhile to mention that its ΔH_Li-M_′ is still the highest. This result indicates that it is desirable to use Mn_3_(PO_4_)_2_ when its higher reactivity to Li_2_O is required, but the weight of the coating material is not a critical condition to meet. Likewise, W(PO_4_)_2_ is placed 3^rd^ in the descending order of ΔH_Li_ and its corresponding ΔH_Li_ with respect to the descending order of G_C_ is 2^nd^, which indicates that its reaction with Li_2_O is highly preferable. However, as shown with Mn_3_(PO_4_)_2_, this material requires a large weight because its G_C_ is positioned 8^th^ from the corresponding order of ΔH_Li-M_ and 11^th^ in the descending order of G_C_ values (Table S.3).

As discussed earlier, it is difficult to find coating materials satisfying both criteria of reactivity and gravimetric efficiency. Among 16 calculated materials, Co_3_(PO_4_)_2_ and BPO_4_ reveal moderate capabilities for both constraints. For example, ΔH_Li-M_ of Co_3_(PO_4_)_2_ is ranked 5^th^ and its G_C_ is also positioned high (2^nd^). This means that this MP material can have good performance depending on which constraint is more important. BPO_4_ exhibits modest performance from all perspectives; all of its capabilities are within the 6^th^ place of the 16 MP materials and its capability is not sensitive to the amount used. Its ΔH_Li-M_ value is positioned 6^th^ and its corresponding G_C_ is 5^th^ while its G_C_ is ranked 4^th^ and its corresponding ΔH_Li-M_ is 5^th^.

Since two of the metal elements (Fe and Co) can take multiple oxidation states (*e.g*., Co is in the +2 and +3 states in Co_3_(PO_4_)_2_ and CoPO_4_, respectively), it is important to compare the performances of those species, as shown in the Inset of Fig. 2. In the case of Fe-based phosphate, the comparison shows that Fe_3_(PO_4_)_2_ exhibits better performance than FePO_4_. More specifically, it is more energetically preferable than FePO_4_; its ΔH_Li-M_ value is positioned 2^nd^ while that of FePO_4_ is at the 15^th^ position in the descending order. However, FePO_4_ exhibits better gravimetric efficiency (G_C_ is ranked 5^th^) than that of Fe_3_(PO_4_)_2_ (ranked 8^th^). Hence, one needs to choose Fe-P based on the purpose of use. Reactivities of Co-based phosphates are almost the same (CoPO_4_ and Co_3_(PO_4_)_2_ ranked 4^th^ and 5^th^, respectively) but the G_C_ value of Co_3_(PO_4_)_2_ (ranked 2^nd^) is better than that of CoPO_4_ (ranked 6^th^); hence using Co_3_(PO_4_)_2_ will be more satisfactory.

### Li-removal capacity: simulation vs. experiment

Based on the screened results from the above calculations, we chose four of the 16 MP materials for experimental validation. Fe_3_(PO_4_)_2_, Co_3_(PO_4_)_2_, and Mn_3_(PO_4_)_2_) were chosen because their reaction with Li_2_O is energetically superior to the others; their ΔH_Li-M_ values are all within the top 5 of 16 MP materials for reaction #1. TiPO_4_ was also chosen to verify the general trend of calculated results because its ΔH_Li-M_ is positioned 9^th^.

**Figure 3:**
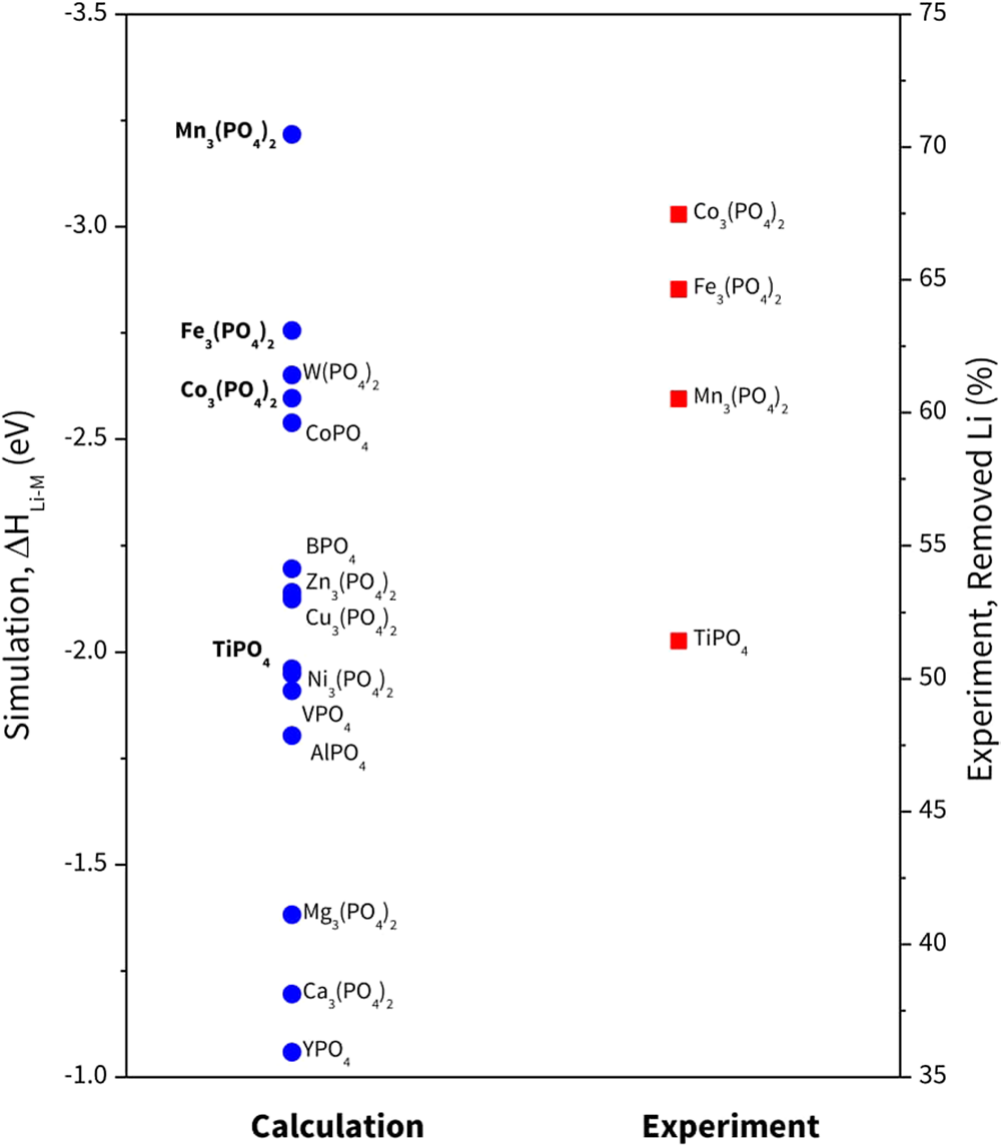
Comparison of Li-removal reactivity from calculations and experiment. Calc denotes that their values are obtained from the descending order of ΔH_Li-M_. MPs enclosed with squares indicate the materials that are compared with experiment.

First, the amounts of Li removed/reacted due to reactions after the Li-reactive coating process were measured (Table S.4). The amounts of LiOH and Li_2_CO_3_ were measured using titration. This is because during the coating process at 720 °C, these Li compounds are transformed to Li_2_O then they revert to LiOH and Li_2_CO_3_ due to reaction with H_2_O and CO_2_ impurities^17, 27, 28^. The result indicates that Co_3_(PO_4_)_2_ can remove almost 70% of residual Li of the uncoated cathode, and Fe- and Mn-P are also shown to be effective (67% and 64% of Li residues were reduced, respectively). Meanwhile, TiPO_4_ exhibited moderate performance; 51% of Li was removed. We associate this result with the simulations by comparing their ΔH_Li-M_ values from the most preferable reaction of each material (reaction #1, the case for Calc^a^) as shown in Fig. 3. We think this comparison is reasonable because a larger ΔH_Li-M_ value from simulations means that this reaction is more likely to happen, so it can remove more residual Li during the experiment. The general trend is reasonably in agreement, *i.e*., coating materials with better experimental Li removal capacities (Co-, Fe-, and Mn-P) also have larger values of ΔH_Li-M_, while TiPO_4_ exhibited poorer performance and has a lower value of ΔH_Li-M_.

### Effect of coating on the electrochemical performance of cathode

**Figure 4:**
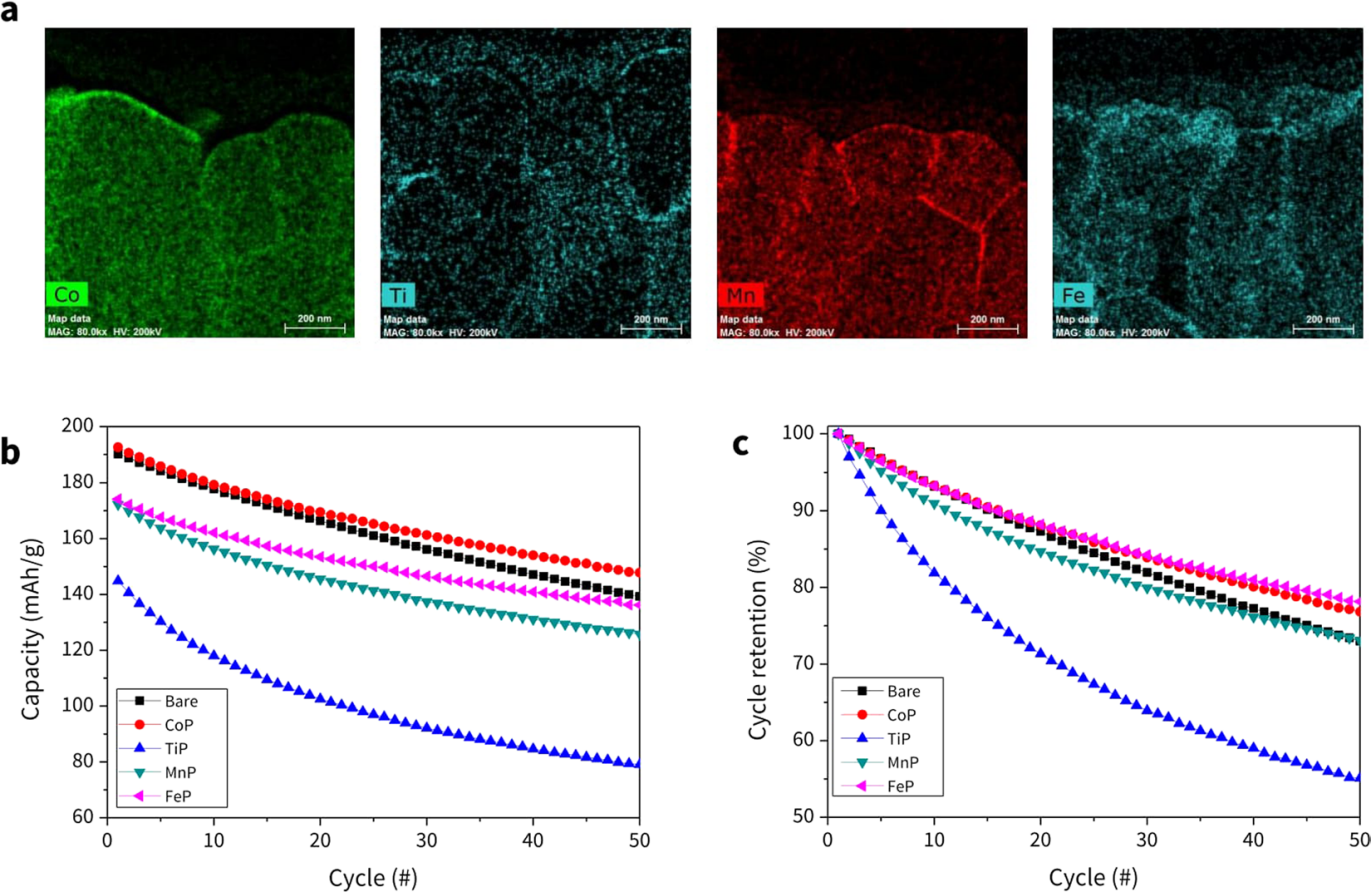
(**a**) EDS elemental mapping of corresponding metal element for Co-, Ti-, Mn-, and Fe-P. (**c**) The variation of the initial capacity and (**d**) the capacity retention rate during 50 cycles for NCM and NCM coated with Co-, Ti-, Mn-, and Fe-P.

The energy dispersive X-ray spectrometric (EDS) elemental maps of the metal elements in the MPs are shown in Fig. 4a. Unlike the pristine structure shown in Figure S.3, it clearly confirms the presence of these coating materials, which are distributed on the surface of the primary and the secondary particles of the NCM. The HAADF (high-angle annular dark-field imaging)-STEM (scanning transmission electron microscopy) images and the quantitative EDS mapping data for all of elements are presented in the SI.

To understand the effect of coating material on the electrochemical performance of NCM cathodes, the Coulombic efficiency, initial capacity, and capacity retention rate (CRR) during cycling were measured (Table 1). The Coulombic efficiency values exhibited by all the coating materials were superior to those of the bare material. Their 1^st^ capacity at 0.2 C rate exhibited that the Co_3_(PO_4_)_2_-coated sample possessed the largest capacity (219.08 mAh/g), even larger than that of the bare (uncoated) material (217.47 mAh/g). This unusual behavior can be attributed to the formation of LiCoO_2_ phase on the surface after reacting with Li compounds, which is suggested from the current simulation result (further discussion will be provided in the following section).

**Table 1:** Initial capacity at 0.1 C, Coulombic efficiency, 2^nd^ capacity at 0.2 C, at 1 C, and the cycle retention rates for uncoated and coated NCM cathodes.

	Initial capacity at 0.1 C (mAh/g)		Coulombic efficiency (%)	2^nd^ capacity at 0.2 C (mAh/g)	1 C capacity (mAh/g)	Cycle retention (%)
Bare (Uncoated)	273.65	223.20	81.56	217.47	189.69	72.98
Co_3_(PO_4_)_2_	243.85	224.53	92.08	219.08	191.53	76.68
TiPO_4_	242.13	217.18	89.69	208.53	143.05	55.12
Mn_3_(PO_4_)_2_	239.14	212.77	88.97	206.92	171.16	73.08
Fe_3_(PO_4_)_2_	238.92	211.64	88.58	205.84	171.9	77.16

Other coating materials had initial capacity reduced by around 10 mAh/g relative to the uncoated case. The capacities of all coated materials dropped significantly (around 30 mAh/g) due to the faster C-rate after measuring the 1^st^ capacity at 1 C rate capacity. This behavior was more severe in the case of TiPO_4_; the capacity decreased from 208.53 to 143.05 mAh/g after changing the rate from 0.2 C to 1 C.

The trend and values of capacity and CRR for all materials up to 50 cycles with 1 C rate are presented in Fig. 4b,c and Table 1. Co_3_(PO_4_)_2_ coating exhibited the best performance considering its 1^st^ cycle capacity and the CRR value (76.68%); both properties were larger than those of other materials considered in this study. Fe_3_(PO_4_)_2_ and Mn_3_(PO_4_)_2_ coating materials also demonstrated great performance in terms of CRR (77.16% and 73.08%, respectively), which are larger than that of the uncoated case (72.98%). Although the 1 C capacities with Fe_3_(PO_4_)_2_ and Mn_3_(PO_4_)_2_ coatings are initially smaller than that of the uncoated case, their capacities are expected to be conserved better during cycling considering their CRR values and the trend of curves, compared to that from the uncoated case (Fig. 4b,c). The TiPO_4_ coating exhibits poor performance on all electrochemical properties; its CRR rate is very low (55.12%) and hence, using this material is not desirable. In summary, Co-, Fe-, and Mn-P materials can reduce significant amounts of Li residue and they also exhibit great electrochemical properties as coating materials, while TiPO_4_ is neither effective at removing Li residues nor at conserving the capacity of the cathode during cycling.

### Equilibrium phase verification

To elucidate the equilibrium phase formed on the surface of the cathode after residual Li-removal by coating materials, a simulated experiment was performed by reacting MP materials directly with the Li compounds, followed by comparison of their phases with those obtained in the calculated phase diagram.

**Figure 5:**
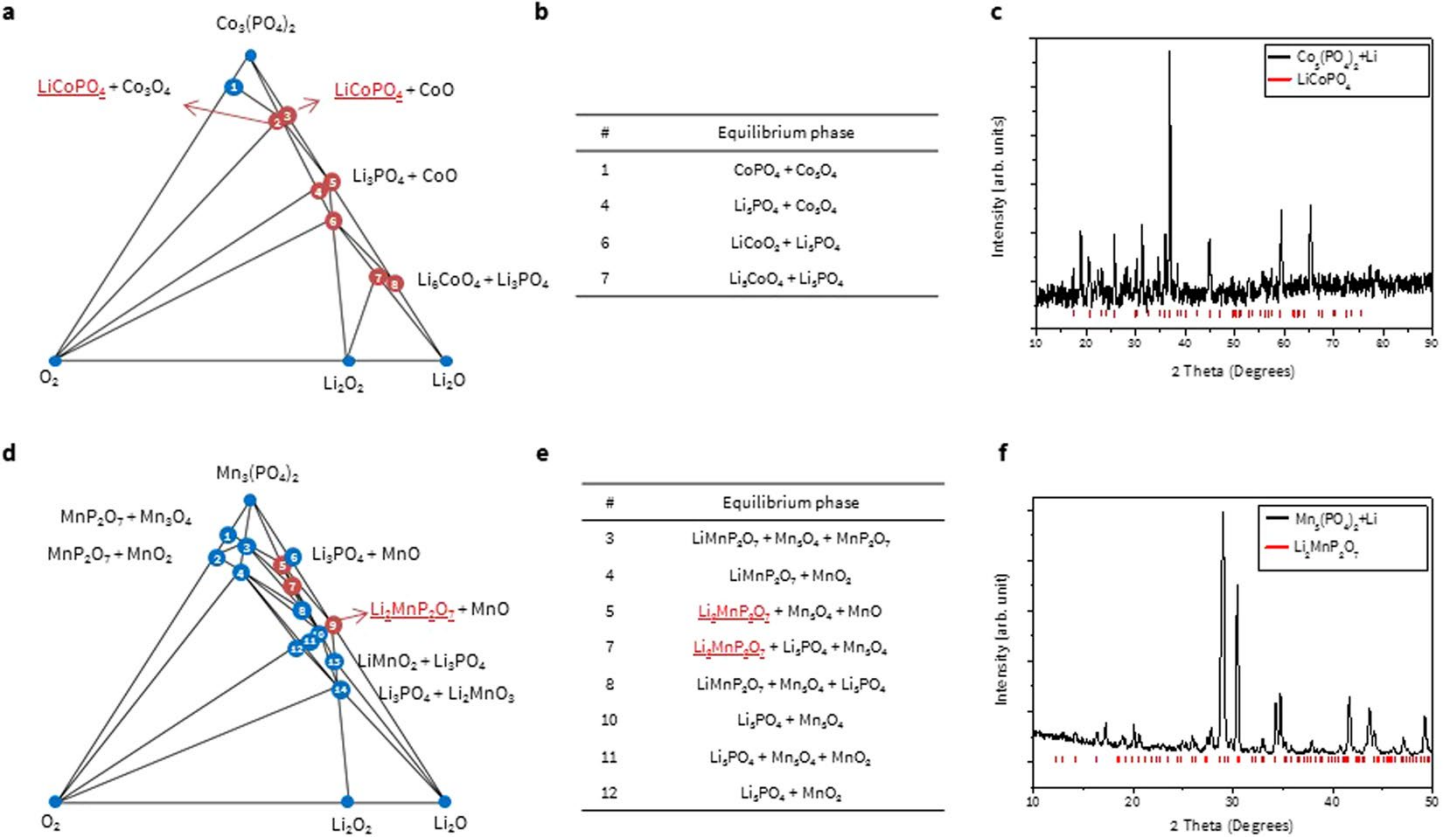
(**a**,**d**) Phase diagram, (**b**,**e**) equilibrium phase information, and (**c**,**f**) XRD pattern of Co_3_(PO_4_)_2_ and Mn_3_(PO_4_)_2_ coating materials after reacting with Li residue (black) and peak information of the discovered phase. Blue and red circles in the phase diagram represent the stable phase and the stable phase confirmed from XRD analysis, respectively.

First, we constructed the Co_3_(PO_4_)_2_ - Li_2_O - O_2_ phase diagram and obtained the reaction product, as shown in Fig. 5a,b. The stable products generated between MP and Li_2_O are important to understand the direct reaction mechanism between the coating materials and residual Li. Since the experiment is usually performed under an O_2_ environment, it is also critical to provide an O_2_ axis and investigate the products that newly emerge. It should be noted that in general more phases are available under O_2_ flow. The complete list of phases available from simulations of other coating materials is tabulated in Table 2.

**Table 2:** Equilibrium phases formed from the reaction between MP-Li_2_O and MP-Li_2_O-O_2_ based on phase diagrams.

Coating Materials	Equilibrium phases	
	MP - Li_2_O	MP - Li_2_O - O_2_
Mn_3_(PO_4_)_2_	Li_2_ MnP_2_ O_7_, Li_3_ PO_4_	LiMnP_2_O_7_, Li_2_ MnP_2_ O_7_, LiMnO_2_, Li_2_ MnO_3_, Li_3_PO_4_
Fe_3_(PO_4_)_2_	Li_2_ Fe_3_ P_4_ O_14_, LiFePO_4_, LiFeO_2_, Li_3_ PO_4_,	LiFe(PO_3_)_4_, Li_2_ Fe_3_ (P_2_ O_7_ )_2_, LiFePO_4_, LiFeP_2_O_7_, LiFeO_2_, Li_2_FeO_3_, Li_5_FeO_4_, Li_3_ PO_4_
W(PO_4_)_2_	Li_4_WO_5_, Li_2_WO_4_, Li_3_PO_4_	n/a
CoPO_4_	LiCoPO_4_,LiCoO_2_, Li_5_CoO_4_, Li_3_PO_4_	n/a
Co_3_(PO_4_)_2_	LiCoPO_4_, Li_6_ CoO_4_, Li_3_ PO_4_	Li_6_ CoO_4_, LiCoPO_4_, LiCoO_2_, Li_5_CoO_4_, Li_3_ PO_4_
BPO_4_	Li_3_B_7_O_12_, Li_2_B_4_O_7_, LiBO_2_, Li_3_BO_3_, Li_3_PO_4_	n/a
Zn_3_(PO_4_)_2_	LiZnPO_4_, Li_10_Zn_4_O_9_, Li_6_ZnO_4_, Li_3_PO_4_	n/a
Cu_3_(PO_4_)_2_	Li_3_ CuO_3_, LiCuO, Li_3_ PO_4_	Li_3_ CuO_3_, Li_3_ PO_4_
TiPO_4_	LiTi_2_ O_4_, Li_2_ TiO_3_, Li_3_ PO_4_	LiTi_2_(PO_4_)_3_, LiTi_2_ O_4_, Li_2_ TiO_3_, LiTi_2_O_4_, Li_4_TiO_4_, Li_3_ PO_4_
Ni_3_(PO_4_)_2_	Li_3_ PO_4_	LiNi_2_O_4_, Li_2_NiO_3_, Li_3_ PO_4_
VPO_4_	LiVP_2_ O_7_, Li_3_ PO_4_	LiVP_2_ O_7_, LiVO_3_, Li_3_VO_4_, Li_3_ PO_4_
AlPO_4_	LiAlO_2_, LiAl_5_O_8_, Li_5_AlO_4_, Li_3_PO_4_	n/a
Mg_3_(PO_4_)_2_	Li_3_PO_4_	n/a
Ca_3_(PO_4_)_2_	Li_3_PO_4_	n/a
FePO_4_	LiFeO_2_, Li_5_ FeO_4_, Li_3_ PO_4_	Li_2_FeO_3_, Li_3_PO_4_, Li_5_ FeO_4_, Li_2_O_2_, Li_3_ PO_4_
YPO_4_	LiYO_2_, Li_3_PO_4_	n/a

Underlined phases denote that they are found both with and without O_2_ environment.

The calculated equilibrium phase of Co_3_(PO_4_)_2_ when the molar ratio of 2:1 is LiCoPO_4_ (reaction #O1), exhibiting good agreement with the experimental result. A previous study also demonstrated that LiCoPO_4_ phase can be formed on the surface of NCA cathode material after Li-reactive reaction with Co_3_(PO_4_)_2_ coating material^16^. LiMnP_2_O_7_ or Li_2_MnP_2_O_7_ phase can be generated from Mn_3_(PO_4_)_2_ coating material after its Li-reactive reaction when the molar ratio is 2:1 or 1:1, respectively; the latter form shows agreement with experiment. Finally, the equilibrium phases can be supported by previous reference in the case of AlPO_4_. When AlPO_4_ is coated on the surface of LCO cathode, it can be transformed to Li_3_PO_4_ and LiAlO_2_ phases due to reaction with Li residues^22^, which agrees with current calculations.

**Figure 6:**
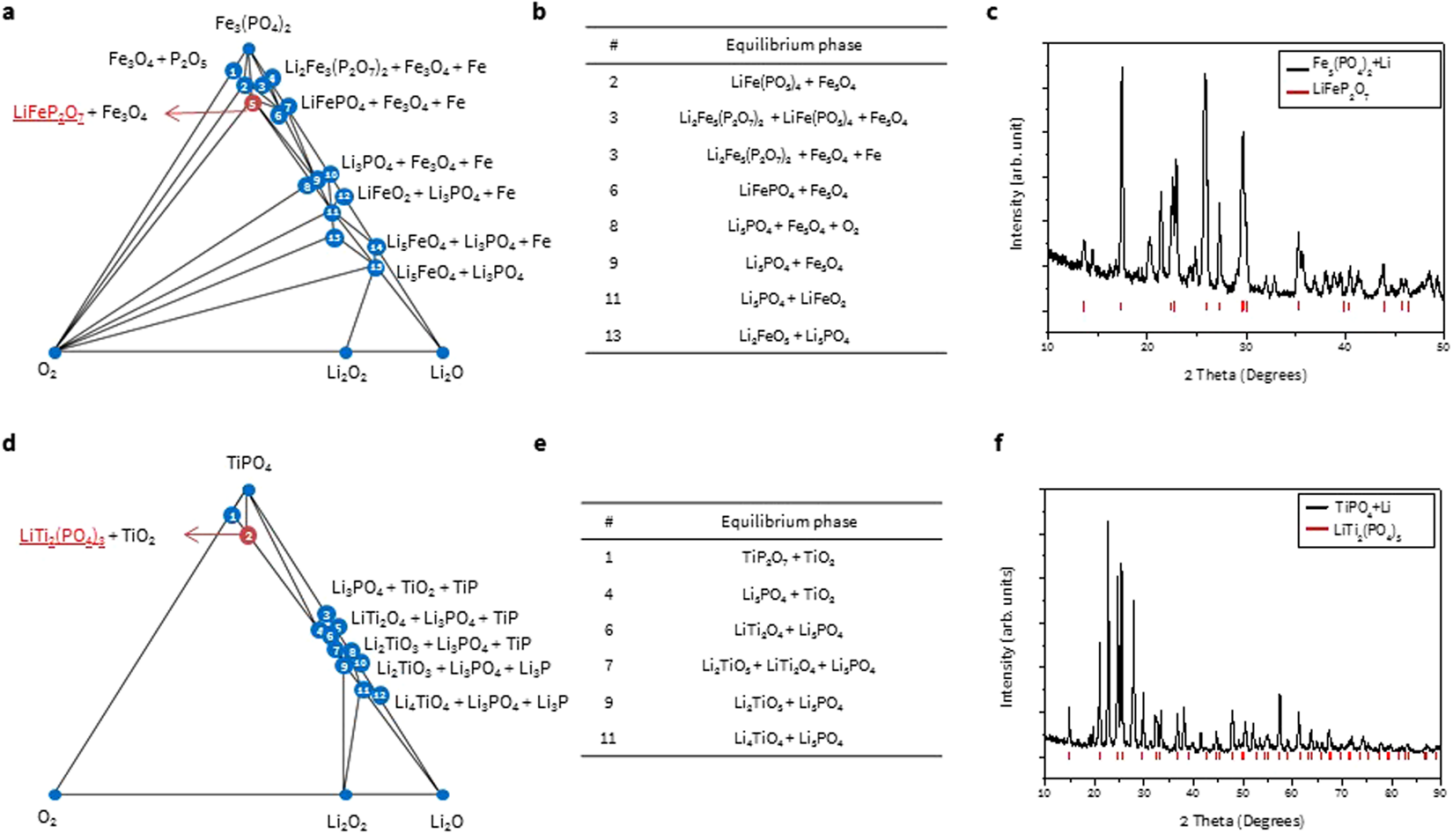
(**a**,**d**) Phase diagram, (**b**,**e**) equilibrium phase information, and (**c**,**f**) XRD pattern of Fe_3_(PO_4_)_2_ and TiPO_4_ coating materials after reacting with Li residue (black) and peak information of the discovered phase. Blue and red circles in the phase diagram represent the stable phase and the stable phase confirmed from XRD analysis, respectively.

Figure 6 provides further validation for the Fe- and Ti-P coating materials. For Fe_3_(PO_4_)_2_, it is important to mention that since the molar ratio of coating materials to residual Li used in the simulated experiment for XRD analysis (molar ratio is 2:1) are much larger than in the actual coating (1 wt% of NCM, molar ratio around 0.3:1), the existing phases after coating can be different. For example, when the molar ratio of 2:1 Fe_3_(PO_4_)_2_ to Li_2_O is provided in a simulated experiment, the possible reaction is #O2 or #O3 in Table S.5, whose equilibrium phase is Li_2_Fe_3_(P_2_O_7_)_2_ or LiFeP_2_O_7_, respectively (the experimentally observed phase is LiFeP_2_O_7_, as shown in Fig. 6c). However, when a smaller molar ratio of coating material is provided, the Li-reacted phase could vary (such as LiFePO_4_, Li_3_PO_4_, Li_2_FeO_3_, etc.) depending on the amount of coating material. Similarly, the possible phases for TiPO_4_ after reaction with Li_2_O can be LiTi_2_(PO_4_)_3_ or Li_3_PO_4_ provided the molar ratio (2:1) is between #O1 (6:1) and #O2 (0.67:1) in Table S.5 (the former phase matches the simulated experiment in Fig. 6f); when a smaller molar ratio is provided, the phase could be Li_3_PO_4_, LiTi_2_O_4_, etc.

In summary, Li-containing phases formed from the simulated experiment were verified by comparing with the phases predicted by calculations with molar ratios based on the phase diagram. This indicates that the phase on the cathode surface can be tuned by altering the amount of coating material with respect to that of the Li residue.

## Conclusions

In this study, we implemented a screening process by constructing phase diagrams based on a first-principles approach to propose the optimal phosphate coating materials that can effectively reduce the amount of residual Li (Li_2_O). Verification of this framework was achieved by performing experiments measuring the remaining Li residue and obtaining electrochemical properties during cycling. Based on the ΔH_Li-M_ values from calculations, the MP coating materials Co_3_(PO_4_)_2_, Mn_3_(PO_4_)_2_, Fe_3_(PO_4_)_2_, and TiPO_4_ were chosen for experimental validation. It was found that the order of reactivity of coating materials for removing Li residues was in good agreement between simulations and experiments; Co-, Mn-, and Fe-P materials exhibited great Li-removal capability. To further confirm the functionalities of coating material on the improvement of cycle life, electrochemical cycling tests showed that Co-, Mn-, and Fe-P materials are effective coating materials for the prevention of capacity fading behaviors. Co_3_(PO_4_)_2_ showed the largest initial capacity among the coating materials tested and its capacity was conserved well, indicating that it would be an ideal coating material for NCM cathode material. In addition, experimental products generated after the reaction of MP and Li were confirmed by comparison with predicted phases obtained from the phase diagram based on calculations.

## Methods

### Computational details: Screening process

We identified the optimal coating materials based on results from first-principles calculations by constructing the phase diagram to obtain Li-containing equilibrium phases. A total of 16 MP materials were calculated with the information of reaction enthalpy (ΔH_Li-M_) and gravimetric capacity (G_C_) when they react with residual Li. ΔH_Li-M_ indicates the reaction energy when reactants are changed to products while G_C_ is the weight of coating material required for the removal of one mole of Li_2_O. Gaseous products generated during this reaction can be disregarded because the Li-reactive coating process occurs at 700 °C; these gases can be removed during ventilation. The overall screening process, computational details, and the database for the formation energy of all materials used in this study are adopted from our previous work^29^. Here is a brief overview of the computational approach.

1. Formation energy values of all related structures were obtained to construct the database. To improve the accuracy of these energy values, density functional theory calculations using the Vienna *ab initio* simulation package (VASP)^30, 31^ with HSE06 hybrid functional were employed^32, 33^.
2. The phase diagrams for MP-Li_2_O-O_2_ were constructed and equilibrium (energetically preferable) phases examined.
3. The reaction equations were obtained based on step 2) with their reaction products and enthalpies.
4. The design chart containing ΔH_Li-M_ and G_C_ information was constructed to identify the ideal MP coating material for removal of Li residue.

Unlike our preceding study, here we focused on the reaction of MP with Li_2_O instead of LiOH and Li_2_CO_3_. This is because the decomposition reactions of both LiOH and Li_2_CO_3_ to Li_2_O could occur beyond the temperature of 700 °C (the coating process in this study was performed at 720 °C), following reactions based on the JANAF-NIMS thermochemistry data table^34^ and post-processed data from our previous study^28^,

1 $$2{\rm{LiOH}}({\rm{s}})\to {{\rm{Li}}}_{2}{\rm{O}}({\rm{s}})+{{\rm{H}}}_{2}{\rm{O}}({\rm{g}})$$

2 $${{\rm{Li}}}_{2}{{\rm{CO}}}_{3}({\rm{s}})\to {{\rm{Li}}}_{2}{\rm{O}}({\rm{s}})+{{\rm{CO}}}_{2}({\rm{g}})$$

LiOH starts to decompose to Li_2_O around 300 °C under average ambient humidity. Decomposition of Li_2_CO_3_ could be dominant below 700 °C with the partial pressure of CO_2_ in the coating environment.

### Experimental methods

#### Sample preparation and coating process

NCM was synthesized by means of a co-precipitation method. Suitable amounts of precursors of Ni, Mn, and Co (Ni:Co:Mn = 91:6:3) were dissolved in deionized (DI) water and stirred to obtain a homogeneous solution. Next, a chelating agent (NH_4_OH) with a stoichiometric amount of NaOH solution was added to achieve co-precipitated (NiMnCo)(OH)_2_ after sufficient stirring. The precipitate was co-ground with a stoichiometric amount of LiOH and calcined at 750 °C under O_2_ flow.

Cobalt nitrate (Co(NO_3_)_3_∙9H_2_O), aluminum nitrate (Al(NO_3_)_3_·9H_2_O), iron(III) nitrate (Fe(NO_3_)_3_·9H_2_O), manganese(II) nitrate tetrahydrate (Mn(NO_3_)_2_·4H_2_O), titanium(IV) oxyacetylacetonate (C_10_H_14_O_5_Ti), and diammonium phosphate ((NH_4_)_2_HPO_4_, termed DMP) were used as source materials to form MP for the surface modification. Assuming the formation of 1 wt% of MP (NCM = 30 g) on the surface of NCM, the amounts of metal and phosphate source were calculated as listed in Table S.1 (Supplementary Information, SI). The coating process was achieved as follows. The stoichiometric metal source was dissolved in DI water. After the NCM powder was suspended in the metal solution, DMP solution was slowly added with a dropper. The solution was then stirred and dried at 120 °C until the solvent evaporated completely. The resulting coated NCM substrate was heated at 720 °C for 5 h in flowing O_2_ gas (30 liter/min).

#### Structural analysis for Li-reactive coating

Simulated experiments were carried out using the same coating materials composed of metal nitrates (the equivalent source as that used for surface coating), DMP, and Li residues (LiOH and Li_2_CO_3_). It was assumed that during the coating process, the Li residues and coating materials (excluding NCM) participated in the reaction. The molar ratio of coating material to Li residue was 2:1. After each material was mixed, the mixture was heated to 720 °C under O_2_ for 5 h.

The material was analyzed after heat treatment using X-ray powder diffraction (XRD) to identify the equilibrium phases in the coating materials. Structural examination of the sample was performed by an X-ray diffractometer using Cu-K_α_ radiation with a scan speed of 0.02° per minute between 10° and 90° at an applied potential of 40 kV and current of 40 mA.

Microstructure analysis was performed using double Cs corrected transmission electron microscopy (TEM, FEI titan cubed 60–300). The composition of the particle surface was confirmed by energy dispersive spectroscopy (EDS, Bruker Super-X).

#### Electrochemical measurements

Composite positive electrodes containing 92 wt% active material, 4 wt% Denka black, and 4 wt% polyvinylidene difluoride (PVdF) were fabricated and pasted on the current collector (aluminum foil). The electrodes were dried at 120 °C under vacuum and then pressed. Metallic lithium was used as the counter electrode. The electrolyte solution consisted of 1.0 M LiPF_6_ dissolved in a solution of fluoroethylene carbonate (FEC) and dimethylene carbonate (DMC). CR2032-type coin cells were assembled in a dry room. The cells were discharged and charged galvanostatically and the measurements were conducted in triplicate at each test condition. The loading level for the active ingredient was 10 mg/cm^2^. The cycling performance of the cells was measured at 25 °C at a charge/discharge rate of 1 C.

The amount of Li residue was estimated by the titration method. Since Li_2_CO_3_ and LiOH are soluble in water, most of the Li sources were assumed to originate from these compounds. The total Li (ppm) was calculated from the following equation.

3 $$\begin{array}{rcl}{\rm{Total\; Li}}({\rm{ppm}}) & = & \frac{2\times {\rm{atomic}}\,{\rm{weight}}\,{\rm{of}}\,{\rm{Li}}}{{\rm{molar}}\,{\rm{weight}}\,{\rm{of}}\,{{\rm{Li}}}_{2}{{\rm{CO}}}_{3}}\times {{\rm{Li}}}_{2}{{\rm{CO}}}_{3}({\rm{ppm}})\\  &  & +\frac{{\rm{atomic}}\,{\rm{weight}}\,{\rm{of}}\,{\rm{Li}}}{{\rm{molar}}\,{\rm{weight}}\,{\rm{of}}\,{\rm{LiOH}}}\times {\rm{LiOH}}({\rm{ppm}})\end{array}$$

#### Data availability

The datasets generated during and/or analyzed during the current study are available from the corresponding author on reasonable request.

## Electronic supplementary material

Supporting Information
