# Supplementary material for: Improved electrochemical properties of LiNi0.91Co0.06Mn0.03O2 cathode material via Li-reactive coating with metal phosphates
Source: Sci Rep. 2017 Aug 2;7:7151. doi: 10.1038/s41598-017-07375-6 (PMC5540909; doi:10.1038/s41598-017-07375-6)
Supplement: Supplementary file 1 — Supporting Information [file 41598_2017_7375_MOESM1_ESM.pdf]

Supporting Information

Improved electrochemical properties of $LiNi_{0.91}Co_{0.06}Mn_{0.03}O_2$ cathode material via Li-reactive coating with metal phosphates

Kyoungmin Min[1,†,*], Kwangjin Park[2,†], Seong Yong Park[1], Seung-Woo Seo[1], Byungjin Choi[2], and Eunseog Cho[1,*]

[1]Platform Technology Lab, Samsung Advanced Institute of Technology, 130 Samsung-ro, Suwon, Gyeonggi-do, 16678, Republic of Korea.

[2]Energy Lab, Samsung Advanced Institute of Technology, 130 Samsung-ro, Suwon, Gyeonggi-do, 16678, Republic of Korea.

†These authors equally contribute to this work

*Corresponding author, E-mail: kmin.min@samsung.com (K. Min), eunseog.cho@samsung.com (E. Cho)

S.1. The amounts of source materials used for coating process.

Coating material	Source	Amount (g)
$Co_3(PO_4)_2$	$Co(NO_3)_3 \cdot 9H_2O$	0.7141
	$(NH_4)_2HPO_4$	0.2160
$TiPO_4$	$C_{10}H_{14}O_5Ti$	0.4504
	$(NH_4)_2HPO_4$	0.3026
$Fe_3(PO_4)_2$	$Fe(NO_3)_3 \cdot 9H_2O$	1.0171
	$(NH_4)_2HPO_4$	0.2216
$Mn_3(PO_4)_2$	$Mn(NO_3)_2 \cdot 4H_2O$	0.6368
	$(NH_4)_2HPO_4$	0.2233

Table S.1. The amount of coating materials (NCM = 30 g).

S.2. Design chart for reactions from MP-Li_2O and MP-Li_2O-O_2

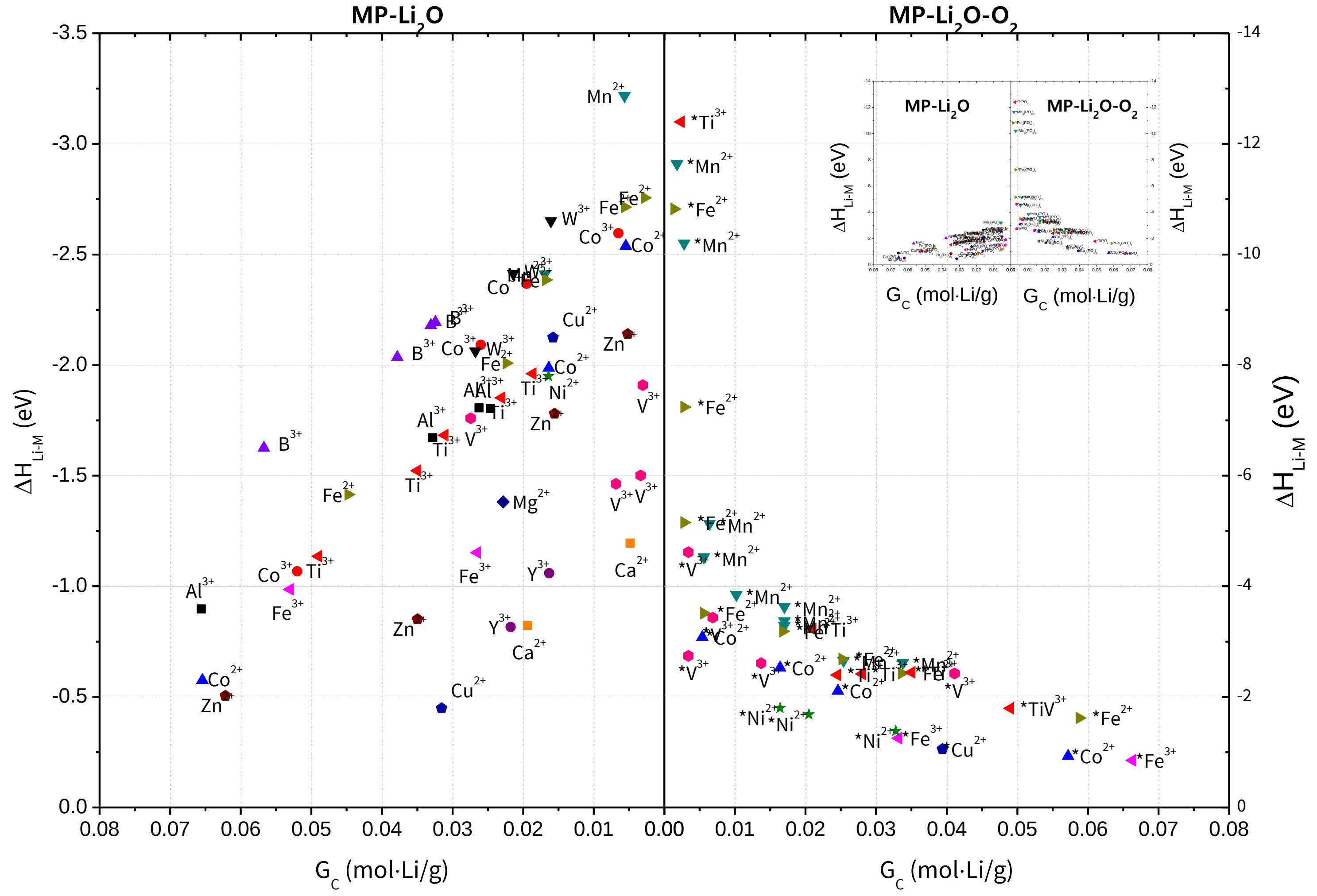

Figure S.1. Design chart for reactions from MP—Li_2O and MP—Li_2O—O_2. (Inset: The y-axis scales were adjusted to equalize the heights of the graphs.)

S.3. ΔH_{LI-M} and G_C values for the $MP-Li_2O-O_2$ reaction

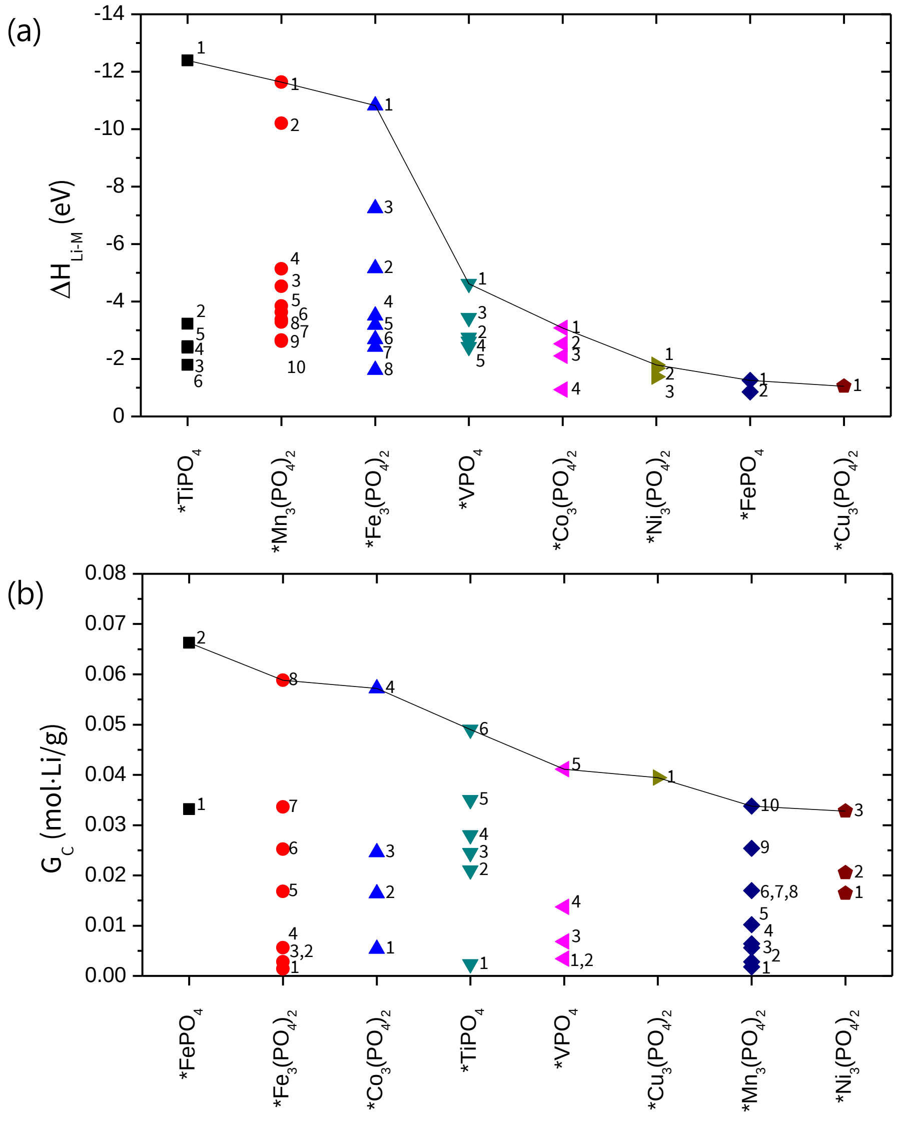

Figure S.2. ΔH_{LI-M} and GC values for the $MP-Li_2O-O_2$ reaction.

Table S.2. Reaction equations for MPO_4-Li_2O.

Eq	Reaction	$\Delta H_{Li\text{-}M}$ (eV)	G_C (mol·Li/g)
$AlPO_4$			
1	$0.67AlPO_4+1.00Li_2O \rightarrow 0.67Li_3PO_4+0.33Al_2O_3$	-1.8033	0.0246
2	$0.63AlPO_4+1.00Li_2O \rightarrow 0.13LiAl_5O_8+0.63Li_3PO_4$	-1.8064	0.0262
3	$0.50AlPO_4+1.00Li_2O \rightarrow 0.50LiAlO_2+0.50Li_3PO_4$	-1.6711	0.0328
4	$0.25AlPO_4+1.00Li_2O \rightarrow 0.25Li_5AlO_4+0.25Li_3PO_4$	-0.8970	0.0656
$CoPO_4$			
1	$2.00CoPO_4+1.00Li_2O \rightarrow 2.00LiCoPO_4+0.50O_2$	-2.5963	0.0064
2	$0.67CoPO_4+1.00Li_2O \rightarrow 0.67Li_3PO_4+0.22Co_3O_4+0.06O_2$	-2.3677	0.0194
3	$0.50CoPO_4+1.00Li_2O \rightarrow 0.50LiCoO_2+0.50Li_3PO_4$	-2.0910	0.026
4	$0.25CoPO_4+1.00Li_2O \rightarrow 0.25Li_5CoO_4+0.25Li_3PO_4$	-1.0678	0.052
$Co_3(PO_4)_2$			
1	$1.00Co_3(PO_4)_2+1.00Li_2O \rightarrow 2.00LiCoPO_4+1.00CoO$	-2.5387	0.0054
2	$0.33Co_3(PO_4)_2+1.00Li_2O \rightarrow 0.67Li_3PO_4+1.00CoO$	-1.9870	0.0164
3	$0.08Co_3(PO_4)_2+1.00Li_2O \rightarrow 0.25Li_6CoO_4+0.17Li_3PO_4$	-0.5756	0.0654
$Mn_3(PO_4)_2$			
1	$1.00Mn_3P_2O_8+1.00Li_2O \rightarrow 1.00Li_2MnP_2O_7+2.00MnO$	-3.2174	0.0056
2	$0.33Mn_3P_2O_8+1.00Li_2O \rightarrow 0.67Li_3PO_4+1.00MnO$	-2.4115	0.017
$FePO_4$			
1	$0.50FePO_4+1.00Li_2O \rightarrow 0.50LiFeO_2+0.50Li_3PO_4$	-1.1517	0.0266
2	$0.25FePO_4+1.00Li_2O \rightarrow 0.25Li_5FeO_4+0.25Li_3PO_4$	-0.9862	0.053
$Fe_3(PO_4)_2$			
1	$2.00Fe_3(PO_4)_2+1.00Li_2O \rightarrow 1.00Li_2Fe_3P_4O_{14}+0.75Fe_3O_4+0.75Fe$	-2.7559	0.0028
2	$1.00Fe_3(PO_4)_2+1.00Li_2O \rightarrow 2.00LiFePO_4+0.25Fe_3O_4+0.25Fe$	-2.7142	0.0056
3	$0.33Fe_3(PO_4)_2+1.00Li_2O \rightarrow 0.67Li_3PO_4+0.25Fe_3O_4+0.25Fe$	-2.3845	0.0168
4	$0.25Fe_3(PO_4)_2+1.00Li_2O \rightarrow 0.50LiFeO_2+0.50Li_3PO_4+0.25Fe$	-2.0090	0.0224
5	$0.13Fe_3(PO_4)_2+1.00Li_2O \rightarrow 0.25Li_5FeO_4+0.25Li_3PO_4+0.13Fe$	-1.4148	0.0448
$Mg_3(PO_4)_2$			
1	$0.33Mg_3(PO_4)_2+1.00Li_2O \rightarrow 0.67Li_3PO_4+1.00MgO$	-1.3818	0.0228
$Zn_3(PO_4)_2$			
1	$1.00Zn_3(PO_4)_2+1.00Li_2O \rightarrow 2.00LiZnPO_4+1.00ZnO$	-2.1397	0.0052
2	$0.33Zn_3(PO_4)_2+1.00Li_2O \rightarrow 0.67Li_3PO_4+1.00ZnO$	-1.7798	0.0156
3	$0.15Zn_3(PO_4)_2+1.00Li_2O \rightarrow 0.11Li_{10}Zn_4O_9+0.30Li_3PO_4$	-0.8505	0.035
4	$0.08Zn_3(PO_4)_2+1.00Li_2O \rightarrow 0.25Li_6ZnO_4+0.17Li_3PO_4$	-0.5049	0.0622
VPO_4			
1	$4.50VPO_4+1.00Li_2O \rightarrow 2.00LiVP_2O_7+0.50V_2PO_5+0.50V_3O_5$	-1.9094	0.003
2	$4.08VPO_4+1.00Li_2O \rightarrow 2.00LiVP_2O_7+0.67V_3O_5+0.08VP$	-1.5009	0.0034
3	$2.00VPO_4+1.00Li_2O \rightarrow 1.00LiVP_2O_7+1.00LiVO_2$	-1.4635	
4	$0.50VPO_4+1.00Li_2O \rightarrow 0.50Li_3PO_4+0.50LiVO_2$	-1.7593	0.0274
$Ni_3(PO_4)_2$			
1	$0.33Ni_3(PO_4)_2+1.00Li_2O \rightarrow 0.67Li_3PO_4+1.00NiO$	-1.9491	0.0164
$Cu_3(PO_4)_2$			
1	$0.33Cu_3(PO_4)_2+1.00Li_2O \rightarrow 0.67Li_3PO_4+1.00CuO$	-2.1247	0.0158

Eq	Reaction	$\Delta H_{Li\text{-}M}$ (eV)	G_C (mol·Li/g)
2	$0.17Cu_3(PO_4)_2+1.00Li_2O \rightarrow 0.25Li_3CuO_3+0.25LiCuO+0.33Li_3PO_4$	-0.4485	0.0316
$Ca_3(PO_4)_2$			
1	$1.33Ca_3(PO_4)_2+1.00Li_2O \rightarrow 0.67Li_3PO_4+1.00Ca_4P_2O_9$	-1.1950	0.0048
2	$0.33Ca_3(PO_4)_2+1.00Li_2O \rightarrow 0.67Li_3PO_4+1.00CaO$	-0.8213	0.0194
YPO_4			
1	$0.67YPO_4+1.00Li_2O \rightarrow 0.67Li_3PO_4+0.33Y_2O_3$	-1.0583	0.0164
2	$0.50YPO_4+1.00Li_2O \rightarrow 0.50LiYO_2+0.50Li_3PO_4$	-0.8156	0.0218
BPO_4			
1	$0.58BPO_4+1.00Li_2O \rightarrow 0.08Li_3B_7O_{12}+0.58Li_3PO_4$	-2.1947	0.0324
2	$0.57BPO_4+1.00Li_2O \rightarrow 0.14Li_2B_4O_7+0.57Li_3PO_4$	-2.1802	0.033
3	$0.50BPO_4+1.00Li_2O \rightarrow 0.50LiBO_2+0.50Li_3PO_4$	-2.0355	0.0378
4	$0.33BPO_4+1.00Li_2O \rightarrow 0.33Li_3BO_3+0.33Li_3PO_4$	-1.6257	0.0568
$W(PO_4)_2$			
1	$0.33W(PO_4)_2+1.00Li_2O \rightarrow 0.67Li_3PO_4+0.33WO_3$	-2.6513	0.016
2	$0.25W(PO_4)_2+1.00Li_2O \rightarrow 0.25Li_2WO_4+0.50Li_3PO_4$	-2.4128	0.0214
3	$0.20W(PO_4)_2+1.00Li_2O \rightarrow 0.20Li_4WO_5+0.40Li_3PO_4$	-2.0635	0.0268
$TiPO_4$			
1	$0.75TiPO_4+1.00Li_2O \rightarrow 0.67Li_3PO_4+0.67TiO_2+0.08TiP$	-1.9609	0.0186
2	$0.61TiPO_4+1.00Li_2O \rightarrow 0.29LiTi_2O_4+0.57Li_3PO_4+0.04TiP$	-1.8524	0.023
3	$0.45TiPO_4+1.00Li_2O \rightarrow 0.40Li_2TiO_3+0.40Li_3PO_4+0.05TiP$	-1.6833	0.0312
4	$0.40TiPO_4+1.00Li_2O \rightarrow 0.40Li_2TiO_3+0.35Li_3PO_4+0.05Li_3P$	-1.5228	0.035
5	$0.29TiPO_4+1.00Li_2O \rightarrow 0.29Li_4TiO_4+0.25Li_3PO_4+0.04Li_3P$	-1.1357	0.049

Table S.3. Descending orders of $\Delta H_{Li\text{-}M}$, G_C', G_C, and $\Delta H_{Li\text{-}M}'$.

Descending order of $\Delta H_{Li\text{-}M}$ and its G_C'			Descending order of G_C and its $\Delta H_{Li\text{-}M}'$		
**Order**	**$\Delta H_{Li\text{-}M}$**	**G_C'**	**Order**	**$\Delta H_{Li\text{-}M}'$**	**G_C**
1	$Mn_3(PO_4)_2$	BPO_4	1	$Mn_3(PO_4)_2$	$AlPO_4$
2	$Fe_3(PO_4)_2$	$FePO_4$	2	$W(PO_4)_2$	$Co_3(PO_4)_2$
3	$W(PO_4)_2$	$AlPO_4$	3	$Ni_3(PO_4)_2$	$Zn_3(PO_4)_2$
4	$CoPO_4$	$Mg_3(PO_4)_2$	4	VPO_4	BPO_4
5	$Co_3(PO_4)_2$	$TiPO_4$	5	BPO_4	$FePO_4$
6	BPO_4	$Ni_3(PO_4)_2$	5	$Fe_3(PO_4)_2$	$CoPO_4$
7	$Zn_3(PO_4)_2$	YPO_4	7	$Mg_3(PO_4)_2$	$TiPO_4$
8	$Cu_3(PO_4)_2$	$W(PO_4)_2$	8	$TiPO_4$	$Fe_3(PO_4)_2$
9	$TiPO_4$	$Cu_3(PO_4)_2$	9	$CoPO_4$	$Cu_3(PO_4)_2$
10	$Ni_3(PO_4)_2$	$CoPO_4$	10	$FePO_4$	VPO_4
11	VPO_4	$Mn_3(PO_4)_2$	11	$AlPO_4$	$W(PO_4)_2$
12	$AlPO_4$	$Co_3(PO_4)_2$	12	$Ca_3(PO_4)_2$	$Mg_3(PO_4)_2$
13	$Mg_3(PO_4)_2$	$Zn_3(PO_4)_2$	13	YPO_4	YPO_4
14	$Ca_3(PO_4)_2$	$Ca_3(PO_4)_2$	14	$Co_3(PO_4)_2$	$Ca_3(PO_4)_2$
15	$FePO_4$	VPO_4	15	$Zn_3(PO_4)_2$	$Mn_3(PO_4)_2$
16	YPO_4	$Fe_3(PO_4)_2$	16	$Cu_3(PO_4)_2$	$Ni_3(PO_4)_2$

Table S.4. The amounts of LiOH, Li_2CO_3, total Li, and removed Li for uncoated and coated NCM cathode.

Coating materials	LiOH (ppm)	Li_2CO_3 (ppm)	Total Li (ppm)	Removed Li (%)
Bare (Uncoated)	6340	11900	4073	
$Co_3(PO_4)_2$	2790	2750	1325	67.46
$Fe_3(PO_4)_2$	3040	2980	1440	64.64
$Mn_3(PO_4)_2$	3780	2730	1608	60.52
$TiPO_4$	3790	4700	1981	51.36

Table S.5. Reaction equations for MPO_4-Li_2O-O_2.

Eq	Reactions	$\Delta H_{Li\text{-}M}$ (eV)	G_C (mol·Li/g)
**$Fe_3(PO_4)_2$**			
O1	$4.00Fe_3(PO_4)_2+1.00Li_2O+2.17O_2\rightarrow3.33Fe_3O_4+2.00LiFeP_4O_{12}$	-10.8247	0.0014
O2	$2.00Fe_3(PO_4)_2+1.00Li_2O+0.50O_2\rightarrow1.00Fe_3O_4+1.00Li_2Fe_3P_4O_{14}$	-5.1537	0.0028
O3	$2.00Fe_3(PO_4)_2+1.00Li_2O+1.17O_2\rightarrow2.00LiFeP_2O_7+1.33Fe_3O_4$	-7.2424	0.0028
O4	$1.00Fe_3(PO_4)_2+1.00Li_2O+0.17O_2\rightarrow0.33Fe_3O_4+2.00LiFePO_4$	-3.5135	0.0056
O5	$0.33Fe_3(PO_4)_2+1.00Li_2O+0.50O_2\rightarrow0.67Li_3PO_4+0.33Fe_3O_4+0.33O_2$	-3.1838	0.0168
O6	$0.22Fe_3(PO_4)_2+1.00Li_2O+0.17O_2\rightarrow0.44Li_3PO_4+0.67LiFeO_2$	-2.6831	0.0252
O7	$0.17Fe_3(PO_4)_2+1.00Li_2O+0.25O_2\rightarrow0.33Li_3PO_4+0.50Li_2FeO_3$	-2.4251	0.0336
O8	$0.10Fe_3(PO_4)_2+1.00Li_2O+0.07O_2\rightarrow0.19Li_3PO_4+0.29Li_5FeO_4$	-1.6189	0.0588
**$Co_3(PO_4)_2$**			
O1	$1.00Co_3(PO_4)_2+1.00Li_2O+0.17O_2\rightarrow0.33Co_3O_4+2.00LiCoPO_4$	-3.0809	0.0054
O2	$0.33Co_3(PO_4)_2+1.00Li_2O+0.17O_2\rightarrow0.67Li_3PO_4+0.33Co_3O_4$	-2.5293	0.0164
O3	$0.22Co_3(PO_4)_2+1.00Li_2O+0.17O_2\rightarrow0.44Li_3PO_4+0.67LiCoO_2$	-2.1065	0.0246
O4	$0.10Co_3(PO_4)_2+1.00Li_2O+0.07O_2\rightarrow0.19Li_3PO_4+0.29Li_5CoO_4$	-0.9283	0.0572
**$TiPO_4$**			
O1	$6.00TiPO_4+1.00Li_2O+1.50O_2\rightarrow2.00LiTi_2P_3O_{12}+2.00TiO_2$	-12.3962	0.0024
O2	$0.67TiPO_4+1.00Li_2O+0.17O_2\rightarrow0.67Li_3PO_4+0.67TiO_2$	-3.2239	0.021
O3	$0.57TiPO_4+1.00Li_2O+0.07O_2\rightarrow0.29LiTi_2O_4+0.57Li_3PO_4$	-2.3937	0.0246
O4	$0.50TiPO_4+1.00Li_2O+0.08O_2\rightarrow0.17Li_2TiO_3+0.17LiTi_2O_4+0.50Li_3PO_4$	-2.4135	0.028
O5	$0.40TiPO_4+1.00Li_2O+0.10O_2\rightarrow0.40Li_2TiO_3+0.40Li_3PO_4$	-2.4411	0.035
O6	$0.29TiPO_4+1.00Li_2O+0.07O_2\rightarrow0.29Li_4TiO_4+0.29Li_3PO_4$	-1.7917	0.049
**$Mn_3(PO_4)_2$**			
O1	$3.00Mn_3(PO_4)_2+1.00Li_2O+2.00O_2\rightarrow1.00MnP_2O_7+2.00Mn_3O_4+2.00LiMnP_2O_7$	-11.6344	0.0018
O2	$2.00Mn_3(PO_4)_2+1.00Li_2O+2.50O_2\rightarrow4.00MnO_2+2.00LiMnP_2O_7$	-10.2021	0.0028
O3	$1.00Mn_3(PO_4)_2+1.00Li_2O+0.25O_2\rightarrow1.00Li_2MnP_2O_7+0.50Mn_3O_4+0.50MnO$	-4.5271	0.0056
O4	$0.89Mn_3(PO_4)_2+1.00Li_2O+0.50O_2\rightarrow0.44Li_3PO_4+0.67Mn_3O_4+0.67LiMnP_2O_7$	-5.1342	0.0064
O5	$0.56Mn_3(PO_4)_2+1.00Li_2O+0.22O_2\rightarrow0.33Li_2MnP_2O_7+0.44Mn_3O_4+0.44Li_3PO_4$	-3.8443	0.0102
O6	$0.33Mn_3(PO_4)_2+1.00Li_2O+0.50O_2\rightarrow0.67Li_3PO_4+1.00MnO_2$	-3.6269	0.017
O7	$0.33Mn_3(PO_4)_2+1.00Li_2O+0.17O_2\rightarrow0.67Li_3PO_4+0.33Mn_3O_4$	-3.2847	0.017
O8	$0.33Mn_3(PO_4)_2+1.00Li_2O+0.25O_2\rightarrow0.67Li_3PO_4+0.25MnO_2+0.25Mn_3O_4$	-3.3702	0.017
O9	$0.22Mn_3(PO_4)_2+1.00Li_2O+0.17O_2\rightarrow0.67LiMnO_2+0.44Li_3PO_4$	-2.6576	0.0254
O10	$0.17Mn_3(PO_4)_2+1.00Li_2O+0.25O_2\rightarrow0.33Li_3PO_4+0.50Li_2MnO_3$	-2.6174	0.0338

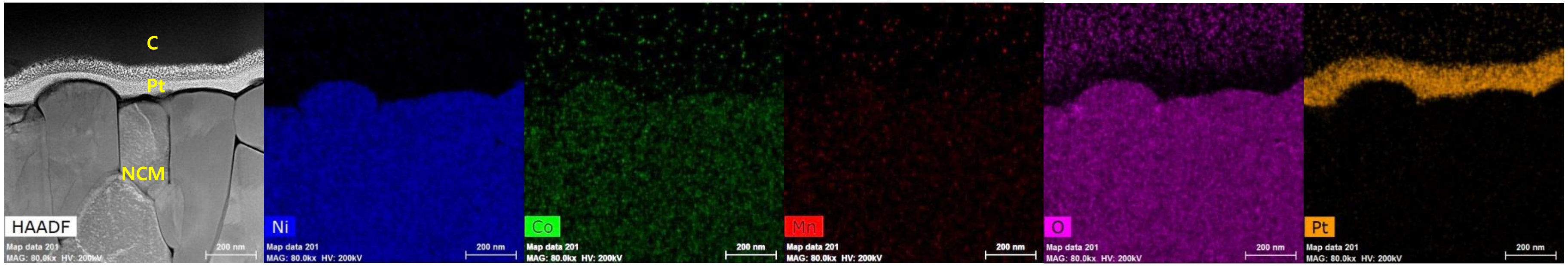

Figure S.3. STEM Z-contrast image of pristine NCM particle (left) and quantitative elemental distribution maps of corresponding area acquired by EDS. Top Pt and C layer were deposited to protect the sample surface during FIB sampling.

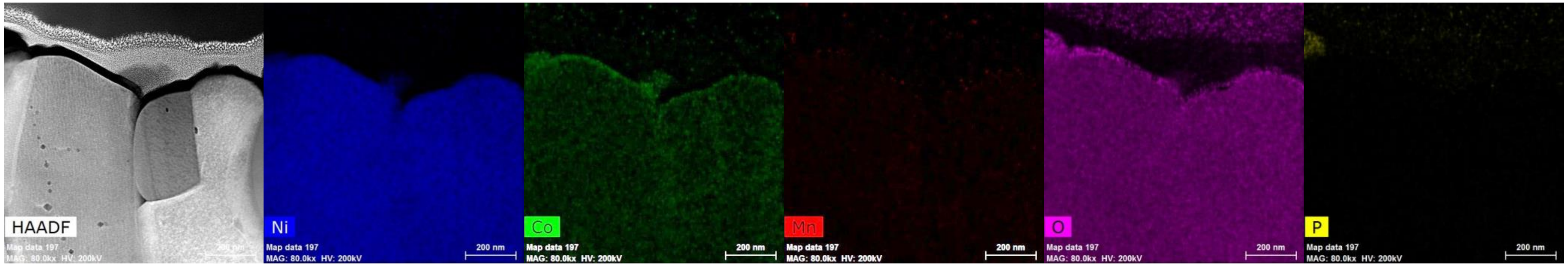

Figure S.4. STEM Z-contrast image of $Co_3(PO_4)_2$-treated NCM particle (left) and elemental distribution maps of corresponding area acquired by EDS.

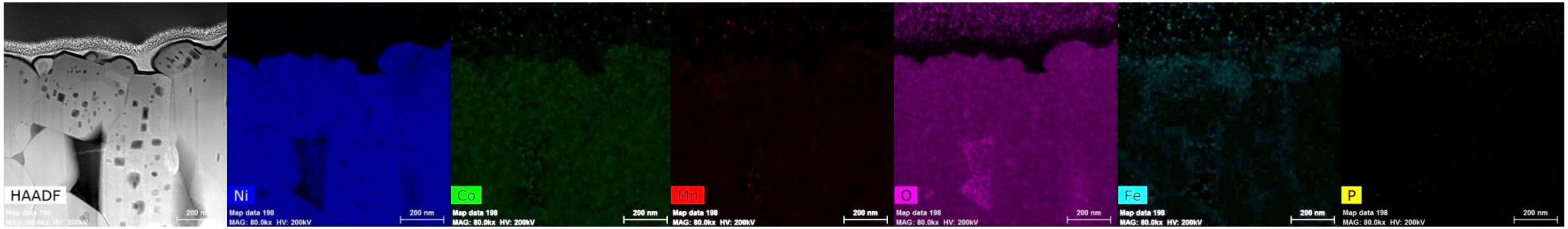

Figure S.5. STEM Z-contrast image of $Fe_3(PO_4)_2$-treated NCM particle (left) and elemental distribution maps of corresponding area acquired by EDS.

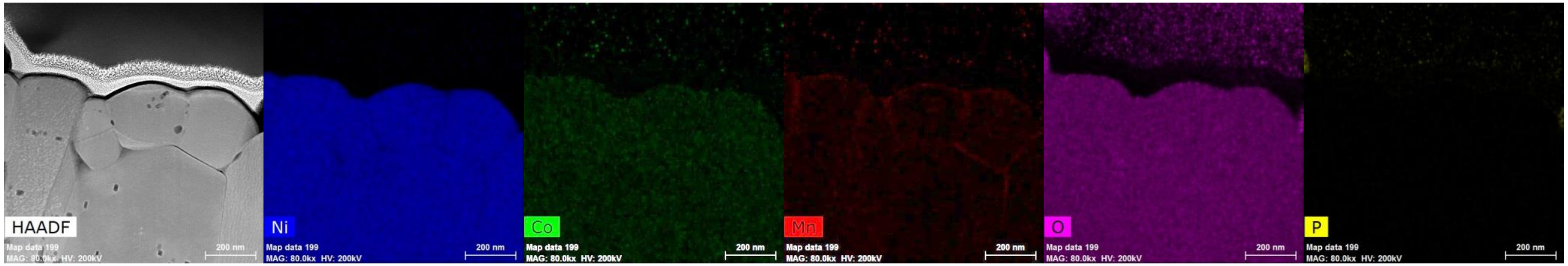

Figure S.6. STEM Z-contrast image of $Mn_3(PO_4)_2$-treated NCM particle (left) and elemental distribution maps of corresponding area acquired by EDS.

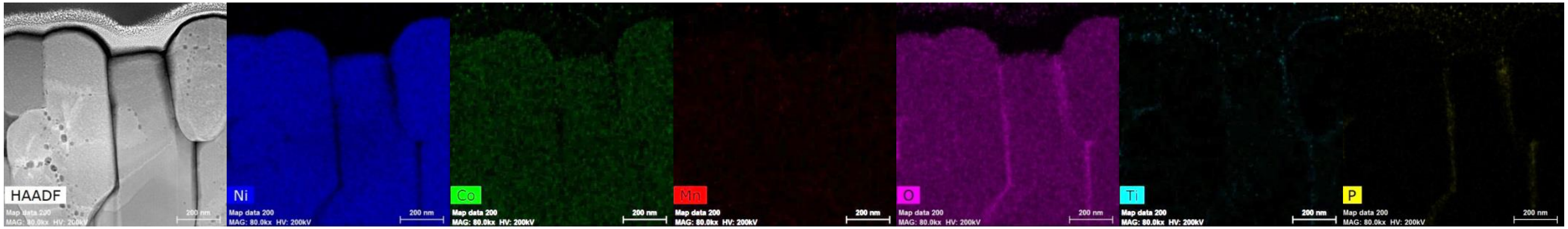

Figure S.7. STEM Z-contrast image of $TiPO_4$-treated NCM particle (left) and elemental distribution maps of corresponding area acquired by EDS.
